# Rat Exterminator

**Published:** 1857-02

**Authors:** 


Rat Exterminator.—It is said that if common cork is sliced as thin as a wafer, and fried in the gravy of meat, but not burnt, and placed where they may be eaten, the rats will soon disappear. We suppose the cork swells and thus destroys.

Snake-Bites.—Since writing our article on insect bites, on page 43, we have noticed that a child was bitten on the arm by a rattle-snake. It was bound up in *wet ashes;* no ill results were observed to follow. Whiskey was swallowed freely. But as spirits have been known to fail signally in such cases, we may attribute the cure to the alkali of the ashes and water.

